# The nitrogen topdressing mode of *indica-japonica* and *indica* hybrid rice are different after side*-*deep fertilization with machine transplanting

**DOI:** 10.1038/s41598-021-81295-4

**Published:** 2021-01-15

**Authors:** Xiaodan Wang, Yaliang Wang, Yuping Zhang, Jing Xiang, Yikai Zhang, Defeng Zhu, Huizhe Chen

**Affiliations:** State Key Laboratory of Rice Biology, China National Rice Research Institute, Hangzhou, 310006 Zhejiang People’s Republic of China

**Keywords:** Ecology, Plant sciences

## Abstract

Determination of the optimal fertilization method is crucial to maximize nitrogen use efficiency and yield of different rice cultivars*.* Side-deep fertilization with controlled-release nitrogen, in conjunction with machine transplanting and subsequent topdressing, was applied to *Indica*–*japonica* hybrid rice ‘Yongyou1540’ (YY1540) and *indica* hybrid rice ‘Tianyouhuazhan’ (TYHZ). Four nitrogen treatments were applied in 2018 and 2019: traditional nitrogen application with quick-release nitrogen (T_1_), single-dose deep fertilization at transplanting with 100% controlled-release nitrogen (T_2_), and deep fertilization of 70% controlled-release nitrogen and topdressing of 30% quick nitrogen at tillering (T_3_), or at panicle initiation (T_4_). Side-deep fertilization reduced the fertilizer application frequency without causing yield loss, T_4_ enhanced the yield of YY1540 by increasing the number of productive tillers and number of spikelets per panicle compared with T_1_, T_2_ and T_3_. The yield of TYHZ showed no significant difference among treatments. The T_4_ treatment decreased the number of tillers at the tilling peak stage and increased the percentage productive tillers and number of differentiated spikelets. Compared with the other treatments, T_4_ increased dry matter accumulation and leaf area index during panicle initiation and grain ripening, and contributed to enhanced nitrogen uptake and nitrogen utilization in YY1540. On average, nitrogen uptake and utilization in YY1540 were highest in T_4_, but no significant differences among treatments were observed in TYHZ. Dry matter accumulation and nitrogen uptake from panicle initiation to heading of YY1540 were correlated with number of spikelets per panicle, but no significant correlations were observed for TYHZ. Supplementary topdressing with quick-release nitrogen at the panicle initiation stage was required to increase yield of *indica*–*japonica* hybrid rice, whereas single-dose deep fertilization with controlled-release nitrogen is satisfactory for the *indica* hybrid cultivar.

## Introduction

Rice is the dominant food crop in China. Generally, more than 50% of the population consumes rice as a staple food. In traditional rice production in China, the amount of nitrogen fertilizer applied is relatively high and the nitrogen use efficiency is low^1^. Traditional fertilization methods typically apply nitrogen fertilizer at three stages: as a basal fertilizer, at the tiller stage, and during panicle initiation. Excessive application of nitrogen fertilizer causes ecological problems, such as soil degradation and pollution^2^, which also influence rice quality by increasing the protein content of grains^3^. In the 1990s, deep placement of nitrogen was reported to enhance nitrogen use efficiency^4^. More recent studies show that deep placement of nitrogen stimulates root growth to increase nutrient uptake, and thereby enhance the early stages of crop growth^5,6^. Under machine transplanting, deep placement of nitrogen fertilizer enhances rice seedling growth and development of more highly productive tillers^7^. In addition, deep application of nitrogen fertilizer has ecological benefits, by reducing emission of greenhouse gases in the paddy field^8,9^, improving soil nitrogen function and the community structure of microorganisms^10^, and maintaining the ecological sustainability of the rice paddy environment.

With the acceleration of urbanization, the rural population is gradually transitioning to cities, therefore rice machine transplanting technology has developed rapidly to overcome the problem of resulting labor shortage^11^. Furthermore, side-deep fertilization simultaneous with machine transplanting was developed, which resulted in large-scale alleviation of the labor shortage, improvement in rice production efficiency, and increase in nitrogen use efficiency^12,13^. With the development of controlled-release fertilizers, side-deep fertilization with machine transplanting has replaced traditional fertilization practices in China in which the quick-release nitrogen fertilizer was used widely.

In China, single season crops accounts for approximately 70% of the rice grown. Hybrid rice comprises 50% of rice production in China, which produces higher yields owing to the strong tillering ability and large spikelets^14^. In recent years, *indica/japonica* hybrid rice has been widely grown in the middle and lower reaches of the Yangtze River of China. These cultivars produce significantly improved yields on account of the larger panicles and longer growth season compared with typical indica hybrid rice^15,16^. The nitrogen fertilizer application rate is important for dry matter accumulation, a high application rate is necessary in *indica–japonica* hybrid rice to obtain a higher yield than that attained with inbred hybrid rice^17,18^. In particular, nitrogen application must be sufficient to meet the demand for spikelet differentiation and the longer grain-ripening period^19^.

Controlled-release fertilizer reduces nitrogen loss. Single-dose deep fertilization with controlled-release nitrogen fertilizer and machine transplantation improves rice yield and nitrogen use efficiency in double-season rice production^20^. Owing to differences in cultivar characteristics, a single application of slow-release fertilizer seems inadequate to fulfill the nitrogen needs of some high-yielding cultivars^12^, which is caused by mismatch between the growth period and fertilizer release. However, differences in the response to side-deep fertilization with machine transplanting between *indica*–*japonica* hybrid and *indica* hybrid rice are unclear.

In 2018, the *indica*–*japonica* hybrid rice ‘Yongyou1540′ (YY1540) and *indica* hybrid rice ‘Tianyouhuazhan’ (TYHZ) were subjected to ten treatments to study differences in the response of *indica-japonica* hybrid rice and *indica* hybrid rice to controlled-release nitrogen application.. Nitrogen topdressing at panicle initiation was beneficial for yield formation in YY1540, whereas a single-dose nitrogen application satisfied the nitrogen needs of TYHZ. The nitrogen application ratio of 70% controlled-release nitrogen applied as side-deep fertilization with machine transplanting to 30% quick-release nitrogen applied as a topdressing at panicle initiation induced the highest yield-increasing effects in YY1540 (Table S1.). Therefore, in 2019, we repeated the experiment with four nitrogen application treatments, consisting of traditional nitrogen application (T_1_), a single-dose controlled-release nitrogen application (T_2_), and 70% controlled-release nitrogen as side-deep fertilization with machine transplanting and 30% as topdressing applied at the tillering stage (T_3_) or the panicle initiation stage (T_4_). The aim was to ascertain the difference in response to nitrogen fertilization mode between the large-panicle *indica*–*japonica* hybrid rice and medium-panicle *indica* hybrid rice, elucidate the factors that contributed to the response, and propose an appropriate controlled-release nitrogen application mode to accompany machine transplanting for *indica–japonica* hybrid rice and *indica* hybrid rice.

## Results

### Yield and yield components

The change in grain yield of the two the cultivars differed in response to controlled-release nitrogen application (Table 1). In contrast to the traditional fertilization mode (T_1_), single-dose fertilization (T_2_) decreased the yield of YY1540 by 9.5% (*p* > 0.05) in 2018 and slightly increased the yield in 2019, whereas T_2_ slightly increased the yield of TYHZ in 2018 and 2019. The T_4_ treatment increased YY1540 yield by 10.5%, 22.1%, and 17.2% compared with the T_1_, T_2_, and T_3_ treatments in 2018, and by 8.0%, 8.0%, and 18.4% in 2019, respectively, which reflected an increase in spikelet number. The T_3_ treatment caused a reduction in YY1540 yield in 2019. Regarding TYHZ, a slight decrease (*p* > 0.05) in yield was observed under the T_3_ treatment compared with T_1_, T_2_ and T_4_, whereas no significant difference in yield was observed between T_1_, T_2_, and T_4_.

**Table 1:** Effect of nitrogen fertilization mode on grain yield and yield components of *indica–japonica* hybrid rice (‘Yongyou1540’, YY1540) and *indica* hybrid rice (‘Tianyouhuazhan’, TYHZ).

Year	Cultivar	Treatment	The number of productive tillers (10^5^ ha^−1^)	The number of spikelet per panicle	Filled grain rate (%)	Grain weight (mg)	Grain yield (t·ha^−1^)
2018	YY1540	T_1_	15.8 ± 0.1 bc	350.7 ± 6.6 ab	80.9 ± 2.3 a	23.3 ± 0.3 a	10.5 ± 0.3 b
		T_2_	15.3 ± 0.2 c	341.0 ± 32.3 ab	78.3 ± 4.4 a	23.1 ± 0.2 ab	9.5 ± 0.4 c
		T_3_	16.5 ± 1.0 b	327.7 ± 15.4 b	80.0 ± 1.2 a	22.8 ± 0.2 b	9.9 ± 0.7 c
		T_4_	17.5 ± 0.2 a	357.5 ± 10.5 a	80.1 ± 0.3 a	23.1 ± 0.2 a	11.6 ± 0.2 a
	TYHZ	T_1_	19.4 ± 0.3 a	241.0 ± 10.3 a	73.7 ± 1.3 a	22.9 ± 0.4 ab	7.9 ± 0.4 a
		T_2_	19.9 ± 0.4 a	232.1 ± 4.4 a	75.5 ± 1.6 a	23.4 ± 0.5 a	8.1 ± 0.2 a
		T_3_	18.8 ± 0.5 a	239.4 ± 11.8 a	74.9 ± 1.4 a	22.1 ± 0.4 b	7.4 ± 0.4 a
		T_4_	18.6 ± 0.4 a	250.0 ± 10.8 a	76.0 ± 1.4 a	22.4 ± 0.4 b	7.9 ± 0.3 a
2019	YY1540	T_0_	12.4 ± 0.6 b	227.7 ± 17.6 d	82.0 ± 3.3 a	23.3 ± 0.0 a	5.4 ± 0.8 d
		T_1_	16.6 ± 0.9 a	360.7 ± 6.6 b	80.9 ± 3.8 a	23.1 ± 0.1 a	11.3 ± 0.9 b
		T_2_	17.5 ± 0.3 a	352.9 ± 6.4 b	79.0 ± 5.3 a	22.8 ± 0.3 ab	11.3 ± 0.8 b
		T_3_	16.6 ± 1.0 a	327.0 ± 7.7 c	78.3 ± 2.8 a	22.4 ± 0.4 b	10.3 ± 0.5 c
		T_4_	16.9 ± 0.7 a	380.7 ± 12.2 a	81.8 ± 5.1 a	23.5 ± 0.3 a	12.2 ± 1.3 a
	TYHZ	T_0_	16.2 ± 1.0 c	169.1 ± 10.1 b	78.2 ± 3.0 a	23.1 ± 0.3 a	5.0 ± 0.5 c
		T_1_	19.7 ± 0.6 b	233.8 ± 10.8 a	76.7 ± 1.8 a	22.9 ± 0.4 a	8.1 ± 0.4 ab
		T_2_	21.1 ± 0.5 a	237.6 ± 17.7 a	74.9 ± 2.5 a	22.2 ± 0.3 ab	8.3 ± 0.7 a
		T_3_	18.7 ± 0.6 b	234.3 ± 14.8 a	74.9 ± 1.4 a	22.1 ± 0.1 b	7.5 ± 0.3 b
		T_4_	19.3 ± 0.6 b	243.4 ± 10.9 a	75.0 ± 1.0 a	22.9 ± 0.1 a	8.0 ± 0.5 ab
Year			9.96**	1.17	0.09	0.97	9.63**
Cultivar			299.45**	461.09**	34.24**	20.71**	222.00**
Treatment			51.68**	41.99**	1.35	3.37*	72.32**
Year × Cultivar			0.01	3.06	0.01	0.8	4.92*
Year × Treatment			4.91**	0.09	0.35	4.13*	0.9
Cultivar × Treatment			7.06**	4.64**	0.35	0.65	10.32**
Year × Cultivar × Treatment			1.84	0.42	0.64	6.05**	0.69

Values followed by different lower-case letters within the same column are significantly different among treatments. *p < 0.05; **p < 0.01.

**Figure 1:**
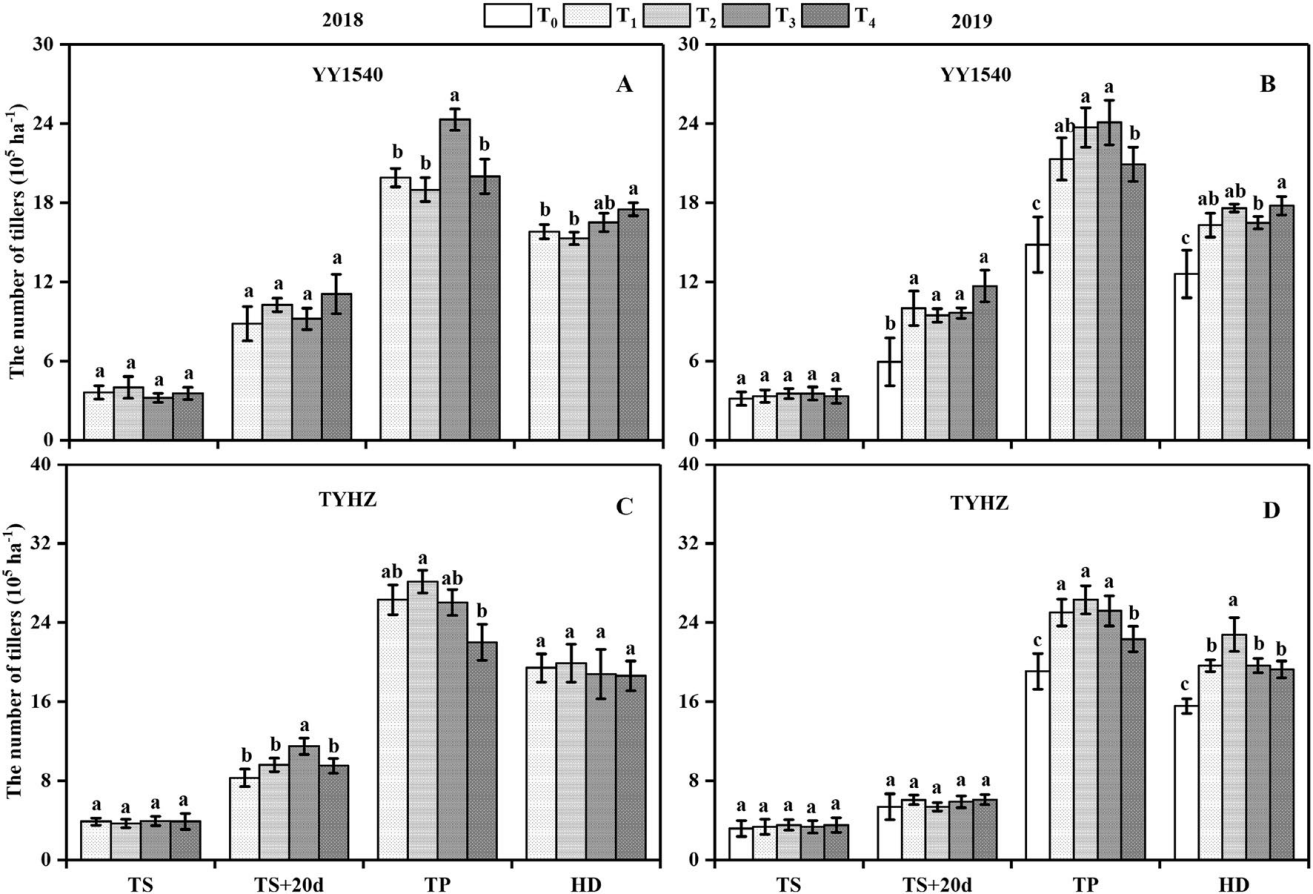
Number of tillers of *indica–japonica* hybrid rice (‘Yongyou1540’, YY1540) and *indica* hybrid rice (‘Tianyouhuazhan’, TYHZ) at four developmental stages under four nitrogen fertilization treatments. (**A**) YY1540 in 2018; (**B**) YY1540 in 2019; (**C**) TYHZ in 2018; (**D**) TYHZ in 2019. Bars with lower-case letters are significantly different at the 0.05 probability level among treatments. *TS* transplanting stage, *TS + 20d* 20 days after transplanting, *TP* tillering peak stage, *HD* panicle heading stage.

**Figure 2:**
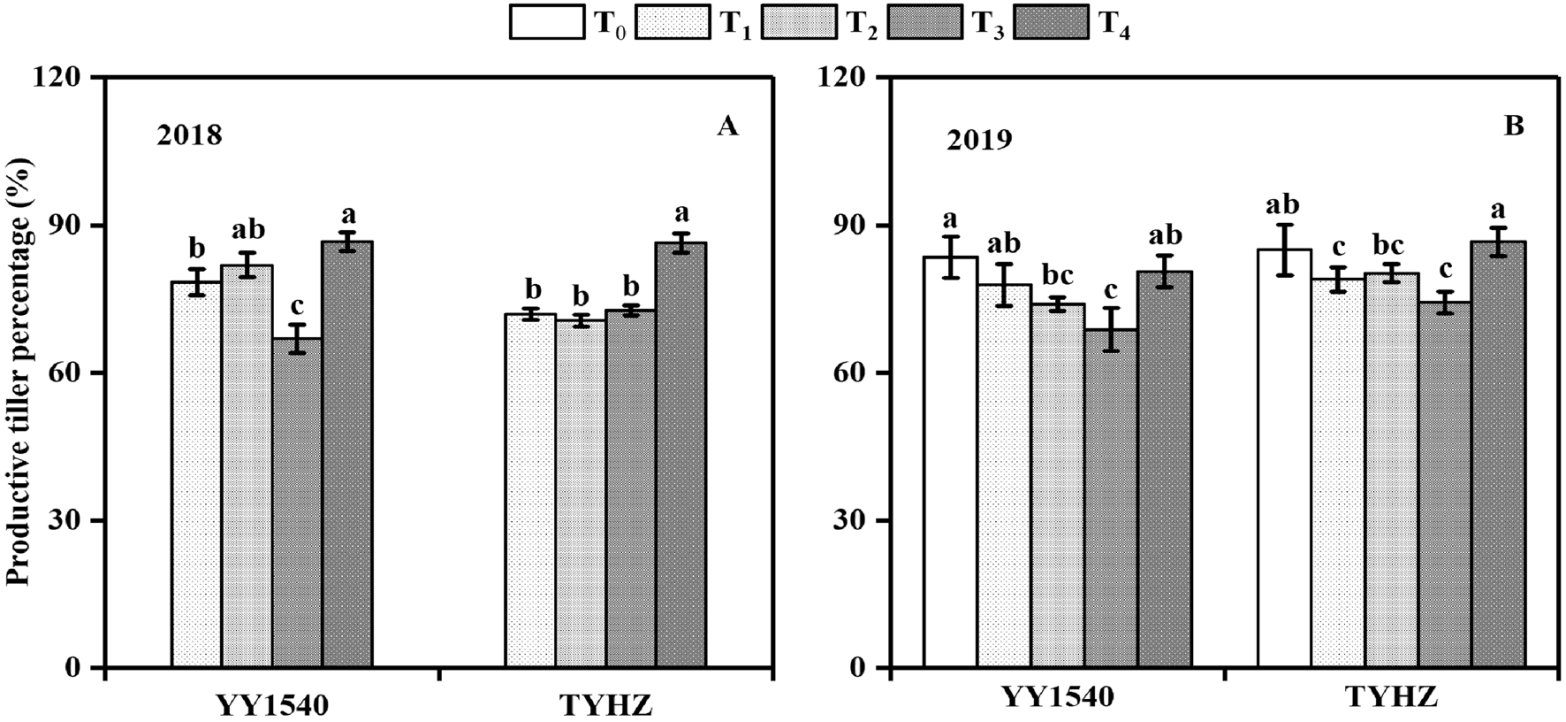
Productive tiller percentage of *indica*–*japonica* hybrid rice (‘Yongyou1540’, YY1540) and *indica* hybrid rice (‘Tianyouhuazhan’, TYHZ) under four nitrogen fertilization treatments. (**A**) 2018; (**B**) 2019. Bars with different lower-case letters are significantly different at the 0.05 probability level among treatments. *T*_*0*_ no-nitrogen application control, *T*_*1*_ traditional nitrogen application, *T*_*2*_ single-dose controlled-release nitrogen application, *T*_*3*_ 70% controlled-release nitrogen as side-deep fertilization with machine transplanting and 30% as topdressing applied at the tillering stage, *T*_*4*_ 70% controlled-release nitrogen as side-deep fertilization with machine transplanting and 30% as topdressing applied at the panicle initiation stage.

Among the different treatments, yield changes depended on the number of productive tillers and number of spikelets per panicle., The highest number of tillers at the tillering peak stage was observed in the T_3_ treatment for YY1540 and T_2_ treatment for TYHZ both in 2018 and 2019 (Fig. 1). However, the highest percentage productive tillers was achieved in the T_4_ treatment in YY1540 and TYHZ except for the no-nitrogen application control (T_0_) in 2019 (Fig. 2). The T_3_ treatment resulted in the lowest productive tillers percentage for YY1540 in 2018 and 2019, whereas no significant difference in productive tillers percentage was observed between T_1_, T_2_, and T_3_ for TYHZ.

With regard to spikelet formation, the main stem was sampled to determine spikelet differentiation and degeneration. The number of spikelets that survived depended primarily on the number of differentiated spikelets. The T_3_ treatment resulted in the minimum number of differentiated spikelets for YY1540 both in 2018 and 2019. The number of differentiated spikelets for YY1540 was highest in the T_4_ treatment, which was 8.6% and 9.8% higher than those of T_1_ in 2018 and 2019, respectively, 18.6% and 20.0% higher than those of T_2_, and 24.3% and 18.7% higher than those of T_3_ in 2018 and 2019, respectively. The T_4_ treatment significantly promoted spikelet degeneration in 2018, but the highest number of spikelets was observed in T_4_ for YY1540. The highest number of differentiated spikelets for TYHZ was observed in the T_4_ treatment, which in 2018 was 1.1%, 4.7%, and 3.0% higher than those in the T_1_, T_2_, and T_3_ treatments, respectively, and in 2019 was 1.3%, 3.0%, and 5.2% higher than those in T_1_, T_2_ and T_3_ treatments, respectively. No significant difference in the proportion of degenerated spikelets was observed among treatments for TYHZ (Table 2).

**Table 2:** Effect of nitrogen fertilization mode on spikelet differentiation and degeneration of primary stems of *indica*–*japonica* hybrid rice (‘Yongyou1540’, YY1540) and *indica* hybrid rice (‘Tianyouhuazhan’, TYHZ).

Year	Cultivar	Treatment	The number of spikelets	The number of differentiated spikelets	The proportion of degenerated spikelets (%)
2018	YY1540	T_1_	396.7 ± 11.5 ab	485.3 ± 9.8 b	18.3 ± 1.8 b
		T_2_	387.5 ± 20.3 bc	444.2 ± 12.6 c	12.8 ± 3.9 c
		T_3_	366.2 ± 10.0 c	423.7 ± 20.3 c	13.5 ± 3.1 c
		T_4_	415.2 ± 9.4 a	526.8 ± 12.0 a	21.1 ± 3.6 a
	TYHZ	T_1_	294.3 ± 9.5 a	368.5 ± 9.3 a	20.1 ± 0.6 a
		T_2_	296.7 ± 23.0 a	356.0 ± 13.3 a	16.6 ± 6.3 a
		T_3_	280.0 ± 6.8 a	361.8 ± 9.2 a	22.6 ± 3.8 a
		T_4_	312.5 ± 27.9 a	372.7 ± 18.5 a	16.3 ± 3.4 a
2019	YY1540	T_0_	244.6 ± 5.2 c	285.4 ± 13.3 c	14.2 ± 2.2 c
		T_1_	396.8 ± 18.5 ab	491.2 ± 32.0 ab	19.2 ± 1.8 b
		T_2_	373.5 ± 19.6 b	449.2 ± 8.0 b	16.9 ± 3.0 bc
		T_3_	369.5 ± 4.4 b	454.2 ± 3.4 b	18.8 ± 0.7 b
		T_4_	406.5 ± 18.3 a	539.2 ± 32.2 a	24.2 ± 1.9 a
	TYHZ	T_0_	200.3 ± 12.9 b	232.4 ± 15.3 b	13.8 ± 0.9 b
		T_1_	295.5 ± 14.3 a	375.2 ± 14.9 a	21.2 ± 1.3 a
		T_2_	298.0 ± 22.4 a	368.8 ± 16.2 a	19.3 ± 2.8 a
		T_3_	289.3 ± 22.1 a	361.3 ± 8.6 a	20.0 ± 1.5 a
		T_4_	307.3 ± 7.0 a	380.0 ± 12.1 a	19.1 ± 1.4 a
Year			417.29**	587.88**	2.37
Cultivar			83.11**	182.79**	8.55
Treatment			0.3	5.56*	7.35*
Year × cultivar			3.72*	18.41**	5.66**
Year × treatment			0.55	0.63	2.12
Cultivar × treatment			0.52	0.19	0.54
Year × cultivar × treatment			0.13	0.97	1.34

Values followed by different lower-case letters within the same column are significantly different among treatments. *p < 0.05; **p < 0.01.

These results indicated that, after side-deep fertilization with controlled-release nitrogen, topdressing at the panicle initiation stage with quick-release nitrogen was necessary to increase yield of the large-panicle *indica*–*japonica* hybrid YY1540. Single-dose side-deep fertilization with machine transplanting was effective for the medium-panicle *indica* hybrid cultivar TYHZ.

### Dry matter accumulation

The highest amount of accumulated total dry matter was observed in the T_4_ treatment for YY1540 in 2018 and 2019, and the lowest total dry matter accumulation was observed in the T_3_ treatment for YY1540 (Table 3). No significant differences were observed among the T_1_, T_2_, T_3_, and T_4_ treatments, although all treatments differed significantly from the T_0_ control for TYHZ in 2019.

**Table 3:** Effect of nitrogen fertilization mode on dry matter accumulation by *indica*–*japonica* hybrid rice (‘Yongyou1540’, YY1540) and *indica* hybrid rice (‘Tianyouhuazhan’, TYHZ).

Year	Cultivar	Treatment	Total dry matter accumulation (t·ha^−1^)	Dry matter accumulation from Sowing to panicle initiation (t·ha^−1^)	Dry matter accumulation from panicle initiation to heading (t·ha^−1^)	Dry matter accumulation from heading to maturing (t·ha^−1^)
2018	YY1540	T_1_	15.7 ± 0.8 a	1.2 ± 0.1 a	7.9 ± 0.8 b	6.7 ± 1.7 a
		T_2_	16.7 ± 0.6 a	1.2 ± 0.1 a	7.4 ± 0.5 b	8.0 ± 0.8 a
		T_3_	13.5 ± 0.3 b	1.1 ± 0.1 a	7.5 ± 1.0 b	4.9 ± 1.0 b
		T_4_	17.8 ± 1.4 a	1.1 ± 0.1 a	8.5 ± 0.3 a	8.2 ± 1.3 a
	TYHZ	T_1_	13.1 ± 0.7 a	1.5 ± 0.2 a	8.0 ± 0.6 a	3.5 ± 0.5 a
		T_2_	12.9 ± 0.3 a	1.5 ± 0.1 a	8.0 ± 0.7 a	3.4 ± 0.6 a
		T_3_	13.2 ± 0.6 a	1.5 ± 0.1 a	8.0 ± 0.6 a	3.7 ± 0.3 a
		T_4_	13.4 ± 0.8 a	1.5 ± 0.2 a	7.8 ± 0.3 a	4.1 ± 0.6 a
2019	YY1540	T_0_	13.0 ± 0.4 c	0.6 ± 0.1 b	6.9 ± 1.1 c	5.5 ± 0.7 c
		T_1_	15.8 ± 0.1 b	1.1 ± 0.0 a	9.1 ± 0.6 a	5.7 ± 0.6 bc
		T_2_	17.4 ± 0.5 b	1.1 ± 0.0 a	8.9 ± 2.0 bc	7.5 ± 1.8 b
		T_3_	15.2 ± 0.5 b	1.1 ± 0.0 a	8.1 ± 0.2 ab	5.9 ± 0.6 c
		T_4_	19.6 ± 1.2 a	1.0 ± 0.0 a	8.9 ± 0.4 a	9.4 ± 1.4 a
	TYHZ	T_0_	9.4 ± 0.9 b	0.9 ± 0.1 b	8.0 ± 0.6 ab	0.5 ± 0.6 b
		T_1_	12.0 ± 0.8 a	1.2 ± 0.1 b	8.2 ± 0.5 ab`	2.6 ± .1.1 a
		T_2_	12.5 ± 1.0 a	2.0 ± 0.2 a	7.9 ± 0.2 b	2.6 ± 1.1 a
		T_3_	13.1 ± 0.2 a	1.3 ± 0.2 b	9.3 ± 0.8 a	2.5 ± 0.8 a
		T_4_	13.7 ± 1.1 a	1.5 ± 0.1 b	8.5 ± 1.1 ab	3.7 ± 1.2 a
Year			205.70**	143.36**	0.31	208.73**
Cultivar			35.58**	33.23**	2.38	13.09**
Treatment			2.08	2.08	8.01**	2.42
Year × cultivar			9.14**	5.36**	1.85	2.87
Year × treatment			7.25*	0.8	0.50	4.88*
Cultivar × treatment			1.63	5.88**	0.09	1.1
Year × cultivar × treatment			0.07	7.76**	1.10	1.73

Values followed by different lower-case letters within the same column are significantly different among treatments. *p < 0.05; **p < 0.01.

Significant differences in dry matter accumulation for YY1540 were observed between the panicle initiation and heading stages and between the heading and maturity stages. Dry matter accumulation from panicle initiation to heading in the T_4_ treatment was 2.4%, 6.7%, and 11.5% higher than that observed in the T_1_, T_2_, and T3 treatments, respectively, on average in 2018 and 2019. Dry matter accumulation from heading to maturity was 41.9%, 13.5%, and 63.0% higher than that observed in the T_1_, T_2_, and T_3_ treatments, respectively, on average in 2018 and 2019, No difference in dry matter accumulation for TYHZ between the T_1_, T_2_, T_3_, and T_4_ treatments in 2018 and 2019 was observed.

**Figure 3:**
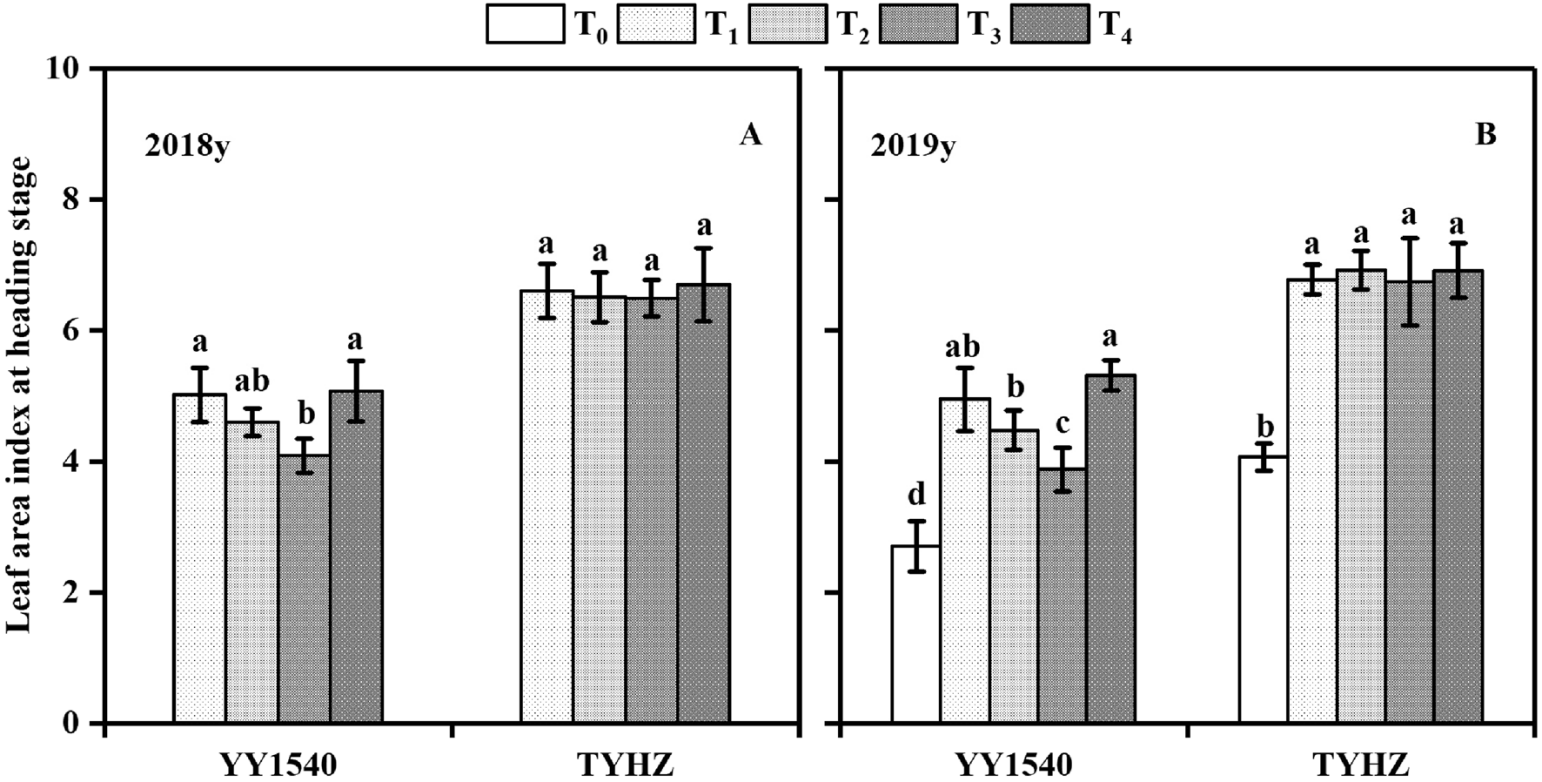
Leaf area index at the heading stage under different treatments. (**A**) 2018; (**B**) 2019. Bars superscripted by different lower-case are significantly different among treatments. *T*_*0*_ no-nitrogen application control, *T*_*1*_ traditional nitrogen application, *T*_*2*_ single-dose controlled-release nitrogen application, *T*_*3*_ 70% controlled-release nitrogen as side-deep fertilization with machine transplanting and 30% as topdressing applied at the tillering stage, *T*_*4*_ 70% controlled-release nitrogen as side-deep fertilization with machine transplanting and 30% as topdressing applied at the panicle initiation stage.

**Figure 4:**
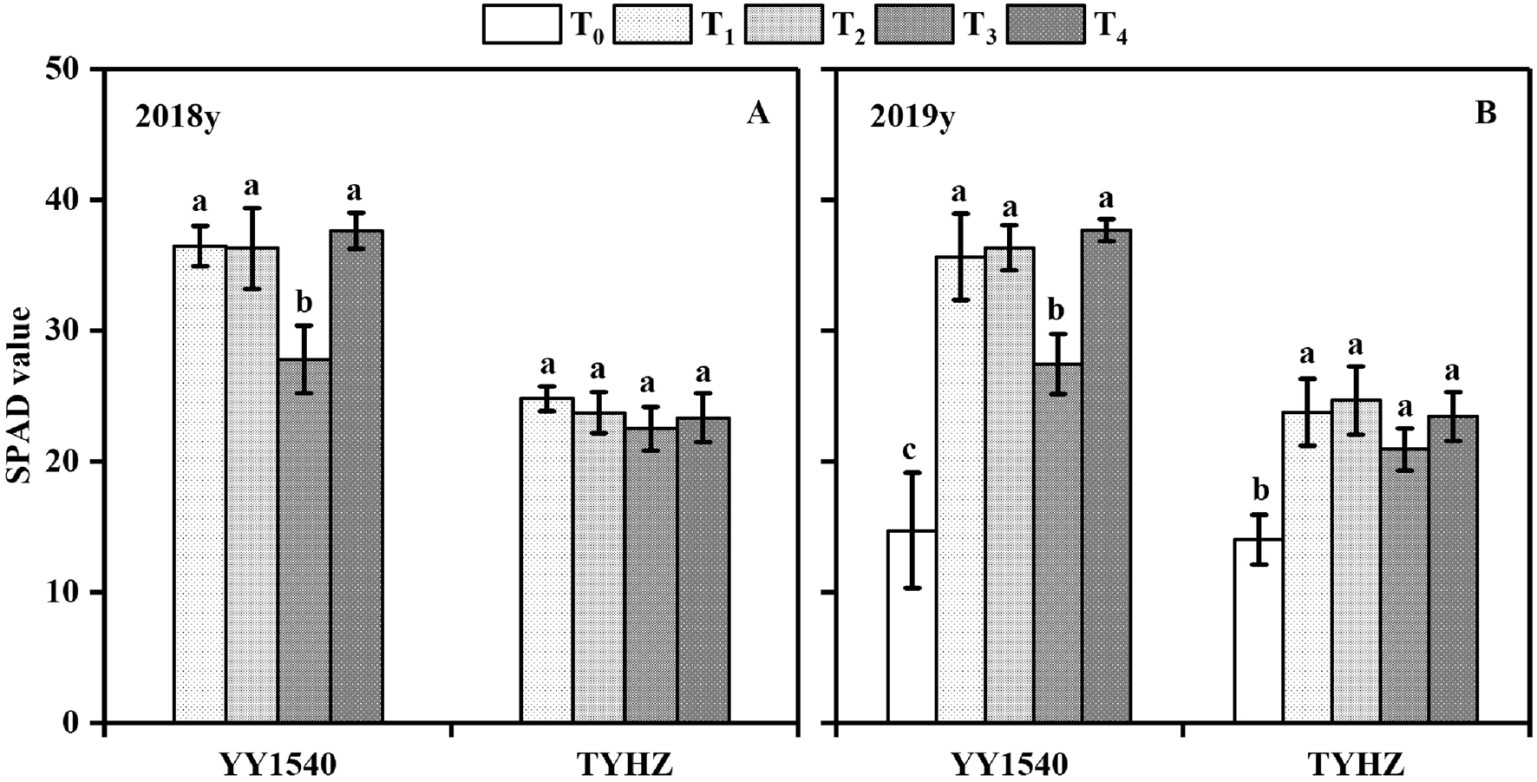
SAPD value at maturing stage of *indica*–*japonica* hybrid rice (‘Yongyou1540′, YY1540) and *indica* hybrid rice (‘Tianyouhuazhan’, TYHZ) under four nitrogen fertilization treatments. (**A**) 2018; (**B**) 2019. Bars with different lower-case are significantly different at the 0.05 probability level among treatments. *T*_*0*_ no-nitrogen application control, *T*_*1*_ traditional nitrogen application, *T*_*2*_ single-dose controlled-release nitrogen application, *T*_*3*_ 70% controlled-release nitrogen as side-deep fertilization with machine transplanting and 30% as topdressing applied at the tillering stage, *T*_*4*_ 70% controlled-release nitrogen as side-deep fertilization with machine transplanting and 30% as topdressing applied at the panicle initiation stage.

Given that the amount of dry matter accumulation depends on leaf photosynthesis activity, we determined that the leaf area index at the heading stage for YY1540 was higher in the T_4_ treatment than in the T_1_, T_2_, and T_3_ treatments in 2018 and 2019, and the T_3_ treatment resulted in the lowest leaf area index. With regard to TYHZ, except for the leaf area index in T_0_, no significant difference in leaf area index was observed between the T_1_, T_2_, T_3_, and T_4_ treatments (Fig. 3). To measure the relative changes in leaf chlorophyll content, the SPAD value at the maturity stage was determined. The SPAD value of the T_3_ treatment was lower than those of the T_1_, T_2_, and T_4_ treatments for YY1540 in 2018 and 2019 (Fig. 4). No significant difference in SPAD value was observed between the T_1_, T_2_, T_3_, and T_4_ treatments, except for the lower SPAD of T_0_ treatment in 2019, for TYHZ.

These results suggested that quick-release nitrogen application at the panicle initiation stage in YY1540 was important for dry matter accumulation from panicle initiation to heading and from heading to maturity. In contrast, single-dose side-deep fertilization with controlled-release nitrogen satisfied the demand for dry matter accumulation in TYHZ.

### Nitrogen accumulation and transport

**Figure 5:**
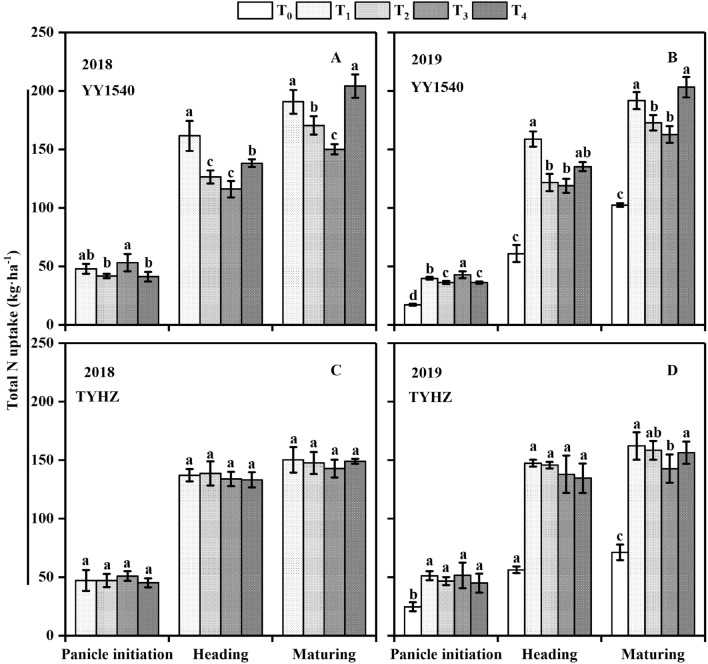
Nitrogen uptake of *indica*–*japonica* hybrid rice (‘Yongyou1540’, YY1540) and *indica* hybrid rice (‘Tianyouhuazhan’, TYHZ) plants under four nitrogen fertilization treatments. (**A**) YY1540 in 2018; (**B**) YY1540 in 2019; (**C**) TYHZ in 2018; (**D**) TYHZ in 2019. Bars with different lower-case letters are significantly different at the 0.05 probability level among treatments. *T*_*0*_ no-nitrogen application control, *T*_*1*_ traditional nitrogen application, *T*_*2*_ single-dose controlled-release nitrogen application, *T*_*3*_ 70% controlled-release nitrogen as side-deep fertilization with machine transplanting and 30% as topdressing applied at the tillering stage, *T*_*4*_ 70% controlled-release nitrogen as side-deep fertilization with machine transplanting and 30% as topdressing applied at the panicle initiation stage.

**Figure 6:**
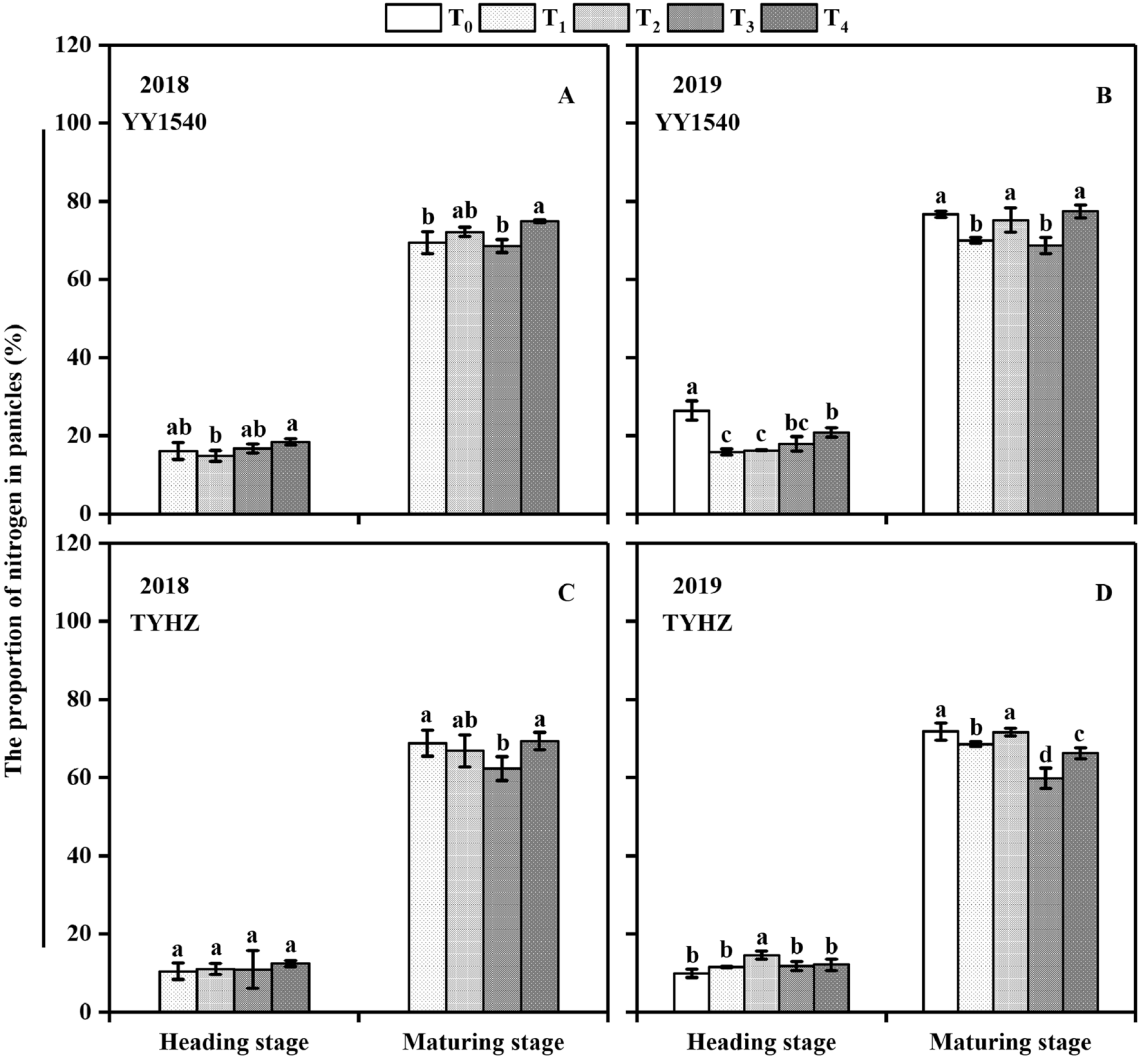
Proportion of nitrogen accumulation in panicles of *indica*–*japonica* hybrid rice (‘Yongyou1540’, YY1540) and *indica* hybrid rice (‘Tianyouhuazhan’, TYHZ) under four nitrogen fertilization treatments. (**A**) YY1540 in 2018; (**B**) YY1540 in 2019; (**C**) TYHZ in 2018; (**D**) TYHZ in 2019. Bars with different lower-case letters are significantly different at the 0.05 probability level among treatments. *T*_*0*_ no-nitrogen application control, *T*_*1*_ traditional nitrogen application, *T*_*2*_ single-dose controlled-release nitrogen application, *T*_*3*_ 70% controlled-release nitrogen as side-deep fertilization with machine transplanting and 30% as topdressing applied at the tillering stage, *T*_*4*_ 70% controlled-release nitrogen as side-deep fertilization with machine transplanting and 30% as topdressing applied at the panicle initiation stage.

Nitrogen accumulation showed a significant difference between the T_1_, T_2_, T_3_, and T_4_ treatments (Fig. 5). Except in T_0_, the nitrogen uptake at the panicle initiation of T_3_ was higher than that of the T_1_, T_2_, and T_4_ treatments for YY1540, the maximum nitrogen uptake was observed in T_1_ in 2018 and 2019 at the heading stage, and the total nitrogen uptake at maturing stage were observed in T_4_ treatment. And in TYHZ, the nitrogen uptake at different developmental stages in 2018 showed no significant differences, whereas significantly lower nitrogen uptake at the maturity stage was observed in T_3_ compared with that in the T_1_, T_2_, and T_4_ treatments. Except for the T_0_ treatment in 2019,, the highest proportion of nitrogen accumulation in panicles at the heading stage for YY1540 was higher in the T_4_ treatment in 2018 and 2019 (Fig. 6A,B). An identical trend was observed at the maturity stage. In contrast, the proportion of nitrogen accumulation in panicles for TYHZ not differ significantly among the treatments at the heading stage in 2018 (Fig. 6C), whereas in 2019, the highest proportion was observed in the T_2_ treatments at the heading and maturity stages disregarding the T_0_ control (Fig. 6D). These results indicated that the T_4_ treatment promoted nitrogen accumulation in YY1540 panicles, whereas the T_2_ treatment fulfilled the panicle nitrogen need demand of TYHZ.

With regard to nitrogen transport TNT, NTE and NCR were significantly higher in TYHZ than in YY1540. The different treatments more strongly influenced TNT, NTE, and NCR in YY1540 than those of TYHZ. The highest TNT was observed in the T_1_ treatment in 2018 and 2019 for YY1540 and in comparison, TNT in the T_2_, T_3_ and T_4_ treatments was 29.3%, 29.7% and 34.7% lower respectively, than that of the T_1_ treatment on average in 2018 and 2019. The highest TNT for TYHZ was observed in the T_4_ treatment, but there were no significant difference observed in among the T_1_, T_2_, T_3_ and T_4_ treatments. The NTE was significantly higher in the T_3_ treatment than that in T_1_, T_2_, and T_4_ in 2018, whereas no significant differences were observed in 2019 for YY1540, and for TYHZ in 2018 and 2019. The T_1_ treatment showed the highest NCR for YY1540 in 2018 and 2019, which was higher than that of T_2_, T_3_, and T_4_ by 22.3%, 14.9%, by 73.0% in 2018, by 42.5%, 10.1%, and 62.0% in 2019, respectively (Table 4). No significant difference was observed among the T_1_, T_2_, T_3_, and T_4_ treatments for TYHZ.

**Table 4:** Nitrogen transport under four nitrogen fertilization treatments in *indica*–*japonica* hybrid rice (‘Yongyou1540’, YY1540) and *indica* hybrid rice (‘Tianyouhuazhan’, TYHZ).

Year	Cultivar	Treatment	TNT (kg·ha^−1^)	NTE (%)	NCR (%)
2018	YY1540	T_1_	77.3 ± 10.6 a	57.0 ± 5.1 b	60.2 ± 3.5 a
		T_2_	60.1 ± 6.3 ab	55.8 ± 4.8 b	49.2 ± 8.0 bc
		T_3_	58.9 ± 4.0 b	61.0 ± 1.0 a	52.4 ± 2.1 b
		T_4_	48.4 ± 6.9 b	42.9 ± 5.8 c	34.8 ± 6.0 c
	TYHZ	T_1_	69.2 ± 7.9 a	55.8 ± 8.5 a	73.7 ± 8.6 a
		T_2_	78.8 ± 9.6 a	59.3 ± 8.9 a	75.7 ± 7.5 a
		T_3_	68.7 ± 7.9 a	61.5 ± 7.3 a	70.7 ± 8.8 a
		T_4_	74.6 ± 6.6 a	62.3 ± 3.4 a	73.2 ± 4.3 a
2019	YY1540	T_0_	32.4 ± 5.1 c	57.1 ± 4.3 a	41.9 ± 7.1 b
		T_1_	76.1 ± 6.9 a	56.9 ± 2.6 a	56.7 ± 4.2 a
		T_2_	48.3 ± 8.4 b	54.3 ± 7.2 a	39.8 ± 8.3 b
		T_3_	49.0 ± 2.3 b	52.1 ± 0.3 a	51.5 ± 2.8 a
		T_4_	51.7 ± 9.3 b	48.2 ± 7.3 a	35.0 ± 6.8 b
	TYHZ	T_0_	30.5 ± 3.9 c	60.3 ± 5.6 a	60.1 ± 9.1 b
		T_1_	79.4 ± 2.9 ab	60.9 ± 1.9 a	71.8 ± 7.5 ab
		T_2_	79.5 ± 3.4 ab	63.9 ± 1.9 a	70.3 ± 6.8 ab
		T_3_	71.2 ± 5.1 b	59.5 ± 12.0 a	83.7 ± 5.6 a
		T_4_	86.7 ± 12.6 a	60.8 ± 4.4 a	85.9 ± 10.4 a
Year			18.28**	8.26**	64.57**
Cultivar			20.42**	1.92	2.59
Treatment			1.27	0.42	1.13
Year × cultivar			6.62**	2.96*	4.06**
Year × treatment			1.4	0.05	0.19
Cultivar × treatment			0.51	1.06	0.8
Year × cultivar × treatment			0.28	0.57	0.72

Values followed by different lower-case letters within the same column are significantly different among treatments.*p < 0.05; **p < 0.01.

*TNT* nitrogen amount transport from stem, sheaths and leaves to panicles, *NTE* nitrogen apparent translocation efficiency of the stem, sheaths and leaves, *NCR* rate of contribution of transferred nitrogen into grains.

### Nitrogen utilization

Based on the nitrogen accumulation analysis in 2018, we added the no-nitrogen treatment (T_0_) in 2019 to calculate nitrogen utilization (Table 5). In YY1540, NMP was highest in T_0_ and in comparison, the NMP was decreased by 31.4%, 25.4%, 26.0%, and 24.4.0% in T_1_, T_2_, T_3_, and T_4_ respectively. The NU_t_E of YY1540 was highest in the T_2_ treatment, but did not differ significantly from T_0_, and that of the T_1_, T_3_ and T_4_ treatments decreased by 9.7%, 4.2%, and 9.5% compared with T_0_. The highest NRF of YY1540 was observed in the T_4_ treatment, which was 11.6%, 30.3% and 40.2% higher than those of the T_1_, T_2_ and T_3_ treatments, respectively. The NAE of the T_4_ treatment was 12.7%, 4.5%, and 32.2% higher than those of T_1_, T_2_, and T_3_ treatments, respectively, for YY1540. In contrast, no significant difference among the T_1_, T_2_, T_3_, and T_4_ treatments were observed for NMP, NU_t_E, NRF, and NAE of TYHZ.

**Table 5:** Nitrogen utilization of in *indica*–*japonica* hybrid rice (‘Yongyou1540’, YY1540) and *indica* hybrid rice (‘Tianyouhuazhan’, TYHZ)under four nitrogen fertilization treatments in 2019.

Cultivar	Treatment	NMP (kg·kg^−1^)	NU_t_E (kg·kg^−1^)	NRF (%)	NAE (kg·kg^−1^)
YY1540	T_0_	127.2 ± 2.0 a	54.9 ± 2.8 ab		
	T_1_	87.3 ± 1.3 c	49.6 ± 3.3 b	45.8 ± 3.8 a	21.4 ± 2.0 b
	T_2_	94.9 ± 5.5 bc	57.2 ± 4.0 a	36.1 ± 3.4 b	23.4 ± 1.9 ab
	T_3_	94.1 ± 6.9 bc	52.6 ± 3.0 ab	31.0 ± 3.6 b	16.6 ± 0.7 c
	T_4_	96.2 ± 3.7 b	49.7 ± 2.0 b	51.8 ± 4.5 a	24.5 ± 0.4 a
TYHZ	T_0_	132.4 ± 3.6 a	74.1 ± 6.1 a		
	T_1_	78.1 ± 3.1 b	48.9 ± 4.4 b	46.7 ± 6.0 a	13.6 ± 0.8 a
	T_2_	78.6 ± 3.2 b	51.4 ± 2.7 b	44.8 ± 4.1 a	14.8 ± 0.7 a
	T_3_	90.3 ± 15.1 b	50.9 ± 4.8 b	36.7 ± 6.2 a	10.2 ± 2.4 a
	T_4_	87.9 ± 87.9 b	51.0 ± 1.6 b	43.7 ± 4.8 a	14.0 ± 1.3 a
Cultivar		8.58**	3.33	0.88	200.68**
Treatment		59.29**	16.56**	11.04**	21.40**
Cultivar × Treatment		2.6	10.35**	3.70*	2.13

Values followed by different lower-case within the same column are significantly different among treatments. *p < 0.05; **p < 0.01.

*NMP* nitrogen dry matter production efficiency, *NU*_*t*_*E* nitrogen utilization efficiency, *NRF* nitrogen apparent recovery fraction, *NAE* nitrogen agronomic efficiency.

### Correlation analysis

To assess the relationship between dry matter accumulation and nitrogen uptake, correlation analysis of the relevant variables at different developmental stages and number of productive tillers, number of spikelets per panicle, or yield was performed on data from 2018 and 2019 combined (Table 6). Under the different nitrogen application treatments, dry matter accumulation from panicle initiation to heading was significantly correlated to the number of spikelets per panicle and yield in YY1540, whereas nitrogen uptake from panicle initiation to heading was significantly correlated to number of spikelets per panicle. However, for TYHZ, no significant correlations were observed between dry matter accumulation and number of productive tillers and number of spikelets per panicle, in the period from sowing to panicle initiation and from panicle initiation to heading, however a significant correlation was observed between the nitrogen uptake from panicle initiation to heading and number of productive tillers and yield. These results suggested the dry matter accumulation and nitrogen uptake from panicle initiation to heading were more important for panicle formation to increase yield in YY1540 than in TYHZ, and that the different fertilization treatments had little effect on TYHZ.

**Table 6:** Correlation analysis between dry matter accumulation and nitrogen uptake in *indica*–*japonica* hybrid rice (‘Yongyou1540’, YY1540) and *indica* hybrid rice (‘Tianyouhuazhan’, TYHZ).

Item	YY1540			TYHZ		
	The number of productive tillers	The number of spikelets per panicle	Theoretical yield	The number of productive tillers	The number of spikelets per panicle	Theoretical yield
Dry matter accumulation from sowing to panicle initiation	− 0.699	− 0.474	− 0.732	0.672	0.187	0.439
Dry matter accumulation from panicle initiation to heading	0.703	0.733*	0.866**	− 0.293	− 0.356	− 0.417
Dry matter accumulation heading to maturing	0.175	0.709*	0.53	− 0.483	0.745*	− 0.183
N uptake from sowing to panicle initiation	− 0.432	− 0.68	− 0.671	− 0.227	− 0.629	− 0.562
N uptake from panicle initiation to heading	− 0.041	0.712*	0.507	0.882*	− 0.301	0.904**
N uptake from heading to maturing	0.592	0.558	0.657	0.109	0.588	0.505

*p < 0.05; **p < 0.01.

## Discussion

### Effect of nitrogen application treatments on yield formation

Compared with the traditional fertilization treatment (T_0_), nitrogen topdressing at the panicle initiation stage (T_4_) resulted in increases in yield and nitrogen utilization efficiency over those attained with controlled-release nitrogen application, The present results verified that application of controlled-release nitrogen can improve the nitrogen utilization efficiency of rice, and that side deep fertilization with machine transplanting significantly improves fertilization efficiency^21,22^. However, the yield under single-dose controlled-release nitrogen application (T_2_) and quick-release nitrogen top dressing at the tillering stage (T_3_) showed a tendency to reduce yield in YY1540 compared with the T_1_ treatment, whereas no significant difference in yield of TYHZ was observed between the T_1_, T_2_, T_3_, and T_4_ treatments, These results suggested that controlled-release nitrogen application did not necessarily promote an increase in yield, and that the advantage of controlled-release nitrogen fertilizer application depended on cultivar characteristics and fertilization methods.

The yield of *indica-japonica* hybrid rice is considerably higher than that of *indica* hybrid rice (Table 1). The principal advantage of the former is the production large panicles under high biomass^23^, which was correlated with the grain yield among the four treatments applied to YY1540^24^. In the present study, the release cycle of the controlled-release fertilizer was about 120 days, Correlation analysis showed that dry matter accumulation and nitrogen uptake from the panicle initiation stage to the heading stage was correlated with spikelet number per panicle in YY1540 (Table 6), which suggested that the slow release of nitrogen by the controlled-release fertilizer did not meet the nitrogen demand of *Indica–Japonica* hybrid rice The productivity advantage of *indica*–*japonica* hybrid rice is dependent on the development of large panicles through enhanced by the spikelet differentiation, which may be associated with the cytokine in synthesis^25,26^. However, in the present study, the nitrogen application rate was 195 kg·ha^−1^, which was slightly lower than the traditional nitrogen application rate used for *indica–japonica* hybrid rice^27^. Whether an increased rate of nitrogen application without topdressing can meet the nitrogen needs for panicle development requires further study, However increased nitrogen application at the tillering stage, as applied in the T_3_ treatment, resulted in a significant reduction in productive tiller percentage and reduced the number of spikelets per panicle, whereas the productive tiller percentage in the T_4_ treatment was significantly increased. Therefore, substantially increased nitrogen application at the tillering stage was not suitable to enhance yield of YY1540. For TYHZ, a slight reduction in yield was also observed in the T_3_ treatment, which was consistent with previous results that postponement of nitrogen topdressing may increase rice yield^28^. In addition, the release of controlled-release nitrogen at two stages is required to meet the nitrogen requirements at different growth stages of *indica–japonica* hybrid rice. For TYHZ, the T_4_ treatment slightly increased (*P* > 0.05) the number of spikelets per panicle, but yield showed no significant difference between the T_1_ and T_4_ treatments owing to the higher tiller number (*P* > 0.05). Thus, a single-dose controlled-release nitrogen application could be adopted for *indica* hybrid rice cultivars such as TYHZ, which are more dependent on the number of productive tillers to attain a high yield than the number of spikelets per panicle^26^.

### Effect of nitrogen application treatments on dry matter accumulation

Dry matter accumulation is significantly influenced by nitrogen application, which plays an important role in maintaining photosynthesis^29^. In the T_3_ treatment, a higher number of tillers were observed at the tillering peak stage compared with that observed in the T_1_, T_2_, and T_4_ treatments in YY1540, which was induced by the excessive nitrogen. Dry matter accumulation from sowing to panicle initiation was not significantly influenced by the nitrogen treatment, but the dry matter accumulation of a single tiller was decreased, which lead to the low percentage productive tillers (Fig. 2), lower leaf area index (Fig. 3), and lower number of spikelets per panicle (Table1) in YY1540 These responses may have been induced by carbohydrate competition between tillers^26,30^. Dry matter accumulation from sowing to panicle initiation was higher in TYHZ than that in YY1540 (Table3), which may explain why the tiller number at the tillering peak stage was higher in TYHZ, This finding may also account for the stronger tillering ability of TYHZ, whereas *indica–japonica* hybrid rice exhibits intersubspecific heterosis for panicle initiation under similar dry matter accumulation from panicle initiation to heading^31,32^.

Nitrogen supplementation is important to maintain carbohydrate supply for grain ripening of *indica–japonica* hybrid rice^19^. In the present study, a larger reduction in grain weight was observed in YY1540 compared with that of TYHZ under the T_3_ treatment. The *indica–japonica* hybrid rice YY1540 has a longer grain-filling stage than that of TYHZ. The significant decrease in dry matter accumulation from heading to maturity may be caused by leaf senescence^4^. The lower SPAD value is consistent with these results (Fig. 4). The SPAD value and dry matter accumulation in the heading-maturity stage were higher in YY1540 than in TYHZ, which indicated that the leaf photosynthetic capacity during the grain-filling stage was higher in YY1540 than in TYHZ. Thus, a large amount of supplementary nitrogen is needed to maintain the chlorophyll content and photosynthesis capacity of YY1540^33^, which was the reason that nitrogen topdressing at panicle initiation stage was needed in YY1540.

### Effect of nitrogen application treatments on nitrogen utilization

Nitrogen accumulation in YY1540 was higher than that in TYHZ (Fig. 5), which is consistent with the stronger nitrogen absorption capacity in *indica–japonica* hybrid rice than that of *indica* hybrid rice^34^. Although the total nitrogen uptake at the heading stage was slightly lower in YY1540 than that in TYHZ, the nitrogen accumulation in panicles was higher in YY1540 than in panicles of TYHZ (Fig. 4), which showed that the contribution of nitrogen to panicle development in YY1540 is greater than that in TYHZ. In TYHZ nitrogen was used mainly for leaf growth, which is consistent with the higher NMP of YY1540 compared with that of TYHZ (Table 5).

The NCR was lower in YY1540 compared with that of TYHZ, and the TNT of YY1540 was highest in the T_1_ treatment (Table 4). These findings are is consistent with a previous study that showed nitrogen accumulation in *indica–japonica* hybrid rice primarily depends on absorption rather than transport^35^, In addition, an adequate late growth nitrogen supply is extremely important for *indica*–*japonica* hybrid rice^27^. The lower NRF on average of YY1540 compared with that of TYHZ also supported this interpretation. However, NRF a lower in the T_2_ and T_3_ treatments than that in the T_1_ and T_4_ treatments (Table 5). Furthermore the NRF of YY1540 in the T_2_ and T_3_ treatments was lower than that in TYHZ, which may be associated with the ability of *indica–japonica* hybrid rice to contribute to nitrogen priming in soil with high microbial activity. The T_2_ treatment resulted in the highest NU_t_E in YY1540 and TYHZ (Table 5). In addition, a reduction in NU_t_E was observed at the quick-release nitrogen application treatments, which showed that controlled-release nitrogen is beneficial for improvement of nitrogen utilization efficiency and reduction of nitrogen losses^36,37^.

## Conclusions

Controlled-release nitrogen side-deep application with machine transplanting can reduce the required fertilization frequency. Nitrogen topdressing at the panicle initiation stage is necessary for high yield formation in *indica*–*japonica* hybrid rice with large panicles. This treatment leads to increase in the number of spikelets per panicle by ensuring sufficient nitrogen fertilizer supply and increase in dry matter accumulation, and results in maximum nitrogen use efficiency in *indica/japonica* hybrid rice. Single-dose controlled-release side-deep fertilization can satisfy the nitrogen absorption demand for high-yield formation in *indica* hybrid rice, which produce medium-type panicles and show enhanced tillering capacity.

## Materials and methods

### Experimental site and meteorological conditions

The field experiments were conducted from May to October in 2018 and 2019 at the China National Rice Research Institute, Hangzhou, Zhejiang Province (119° 55ʹ 48ʺ E, 30° 2ʹ 24ʺ N), China. The experimental field contained common paddy soil with pH 5.45, organic matter 32.13 g·kg^−1^, total nitrogen 1.69 g·kg^−1^, available phosphorus 85.8 mg·kg^−1^ and available potassium 95 mg·kg^−1^. Soil analyses were performed on samples collected from the uppermost 20 cm following the methodology of Ke et al.^38^.

During the 2-year experiment, rainfall, relative humidity, temperature, and solar radiation were measured in the field using a HOBO weather station (MAH-H21, Onset Computer Corporation, Bourne, MA, USA). The average rainfall of 2018 was higher than that of 2019 and the humidity showed a similar trend (Table 7). The monthly average temperature in 2018 ranged from 17.1 °C to 29.4 °C, which was slightly lower than that in 2019 (19.8–32.2 °C). The cumulative solar radiation was 391.7 MJ·m^−2^ in 2019, which was higher than that in 2018 (272.8 MJ·m^−2^).

**Table 7:** Meteorological conditions during the rice growth seasons at the study site.

Year	Month	Rainfall (mm)	Average Temperature (°C)	Average relative humidity (%)	Cumulative solar radiation (MJ/m^2^)
2018	May	129.6	23.5	83.3	202.9
	June	204.0	26.1	84.5	246.7
	July	113.4	29.4	83.7	332.3
	August	201.2	28.3	85.7	268.0
	September	275.6	24.4	86.7	436.5
	October	17.6	17.1	82.1	150.4
2019	May	109.6	21.5	87.3	195.1
	June	185.0	25.8	86.3	235.5
	July	172.9	28.5	82.6	357.0
	August	180.8	29.5	77.3	409.7
	September	36.0	24.7	78.2	325.3
	October	19.7	19.8	80.1	227.6

### Plant material

We selected two super-rice cultivars grown commercially in China. The *indica-japonica* hybrid rice ‘Yongyou1540’ (YY1540) and *indica* hybrid rice ‘Tianyouhuazhan’ (TYHZ), have been widely cultivated in the middle and lower regions of the Yangtze River for single-season rice production for more than 10 years, and are representative of the *indica-japonica* hybrid rice and *indica* rice cultivars in cultivation^39,40^.

The growth period of YY1540 from sowing to maturity in 2018 and 2019 was 161 days and 157 days, respectively. The average spikelet number of YY1540 was 338 per panicle. The growth period of TYHZ from sowing to maturity in 2018 and 2019 was 129 days and 131 days, respectively. The average spikelet number of TYHZ was 217 per panicle. Details of the rice growth period in both years of the study are shown in Table 8.

**Table 8:** Details of the rice growth period in 2018 and 2019.

Cultivar	Year	Sowing date	Transplanting date	Date of panicle initiation beginning	Date of panicle heading beginning	Date of harvest
YY1540	2018	5/17	6/12	7/16	8/22	10/21
	2019	5/17	6/12	7/15	8/22	10/25
TYHZ	2018	5/17	6/12	7/12	8/13	9/25
	2019	5/17	6/12	7/9	8/12	9/23

### Experimental design

The experiment was conducted using a split-plot design with cultivars as primary plots and nitrogen treatments as subplots. A total of 195 kg N ha^−1^ was applied during the rice growth period. The treatments are summarized in Table 9.

**Table 9:** Experimental nitrogen treatments applied in the study.

Treatment	Nitrogen application mode	Conduct year
T_0_	No nitrogen application	2019
T_1_	Traditional nitrogen fertilization with quick-release nitrogen at the 1 days before transplanting (base fertilizer, 40%N), 7 days after transplanting (tillering fertilizer, 30% N) and panicle initiation stage (panicle fertilizer, 30% N)	2018 and 2019
T_2_	Control-release nitrogen by single-dose side deep fertilization with machine transplanting (100% N)	2018 and 2019
T_3_	Controlled-release nitrogen by side deep fertilization machine transplanting (70% N) + Quick-release nitrogen top dressing at 7 days after transplanting (30% N)	2018 and 2019
T_4_	Controlled-release nitrogen by side-deep fertilization machine transplanting (70% N) + Quick-release nitrogen top dressing at panicle initiation stage (30% N)	2018 and 2019

For all treatments, quick-release nitrogen was applied as common urea (nitrogen content: 46%) and controlled-release nitrogen was applied as a slow release fertilizer (N content: 41.6%, Kingenta International Co., Ltd., Shandong, China). Phosphorus fertilizer was applied as calcium superphosphate at the rate of 510 kg·ha^−1^ as a basal dressing. Potassium fertilizer was split-applied with 50% as a basal level and 50% at the panicle initiation stage at a rate of 280 kg·ha^−1^ potassium. The deep fertilization machine used was developed by the Jinhe Agricultural Science and Technology Co, Ltd. (Zhejiang, China) following the methodology of Zhu^20^. The depth of fertilization was 5 cm. The irrigation method followed local practices established to achieve high-yield crops. The treatments were repeated three times. The subplot size was 216 m^2^.

### Determination methods

#### Yield and yield components

Yield and yield components were determined as described by Yoshida^41^. The grain yield was determined from a harvest area of 6 m^2^ in each subplot at the rice brown stage and adjusted to 13.5% grain moisture. The yield components (panicle number, spikelets number per panicle, percentage of filled grains and grain weight) were determined from the plants within a 1 m^2^ area randomly chosen in each subplot (excluding border plants) in accordance with the method of Kamiji^42^. The filled-grain percentage was calculated as follows: number of filled grains per panicle/(number of filled grains per panicle + number of unfilled grains per panicle) × 100.

### Tiller number dynamics

After transplantation, the number of tillers was determined at 7-days intervals. Thirty plants per plot were selected for investigation. The criterion for tiller recognition was presence of three leaves on each tiller. The proportion of effective panicles was calculated as the number of effective panicles/tiller number at the peak stage.

### Spikelet differentiation and degeneration

To maintain a consistent panicle development period for each treatment, ten primary tillers (tagged during rice growth) from each subplot were collected when the panicle was 50% elongated and used to quantify spikelet differentiation and degeneration. The number of degenerated spikelets was calculated by counting the vestiges present on the panicles in accordance with the method of Yao^43^. The number of differentiated spikelets per panicle is the sum of surviving and degenerated spikelets per panicle. The proportion of degenerated spikelets was calculated as follows: number of degenerated spikelets per panicle/number of total differentiated spikelets per panicle × 100.

### Dry matter accumulation, leaf area index and SPAD value

Ten plant hills were sampled from each plot based on the average tiller number at the panicle initiation, heading and maturity stages. To determine level of dry matter accumulation, the sampled plants were dried at 105 °C for 30 min and then dried to a constant weight at 80 °C following the method of Zhu^20^. Panicles, leaves, and stems with leaf sheaths were separated from the plants after the heading stage. An LI-3100C Area Meter (LI-COR, Inc., Lincoln, NE, USA) was used to measure the leaf area of each green leaf. The leaf area per square meter was calculated as the leaf area index following the method of Wu^19^. The SPAD value was determined at the maturity stage using a SPAD chlorophyII meter (SPAD-502PLUS, Spectrum Technologies, Aurora, IL, USA).

### Plant nitrogen content, nitrogen uptake and nitrogen use efficiency

After measurement of the sample dry matter at the panicle initiation, heading and maturity stages, the total nitrogen content of the samples was determined using the micro-Kjeldahl digestion method in accordance to the procedure described by Bremner^44^. To determine the nitrogen content, ground samples (0.20 g) of the panicle, leaf, stem, and sheath were digested in H_2_SO_4_–H_2_O_2_ solution at 420 °C for 2 h and distilled with 10 mol·L^−1^ sodium hydroxide solution. The evaporated NH_3_ was absorbed with Na_2_B_4_O_7_ and titrated with 0.01 mol·L^−1^ sulfuric acid. According to the consumption of the titrant, the percentage of nitrogen was calculated, which was analyzed by the micro-Kjeldahl method (Kjeltec TM 8400, FOSS, Helleröd, Denmark). Nitrogen uptake was calculated using the following formula: dry matter accumulation × nitrogen concentration.

Measures of nitrogen transport and nitrogen use efficiency, namely the amount of nitrogen amount transport from stem, sheaths and leaves to panicles (TNT), apparent nitrogen translocation efficiency of stem, sheaths, and leaves (NTE), rate of contribution of transferred nitrogen into grains (NCR), nitrogen dry matter production efficiency (NMP), nitrogen utilization efficiency (NU_t_E), apparent nitrogen recovery fraction (NRF), and nitrogen agronomic efficiency (NAE) were calculated using the formulas described by López-Bellido et al.

TNT = Nitrogen amount of leaf and stem sheath at the heading stage − Nitrogen amount of leaf and stem sheath at the maturity stage;

NTE = TNT/Nitrogen amount of leaf and stem sheath at the heading stage × 100;

NCR = TNT/Nitrogen amount of grains at the maturity stage × 100;

NMP = Total dry matter accumulation uptake at the maturity stage/Total nitrogen accumulation uptake at the maturity stage;

NU_t_E = Grain yield/Total nitrogen accumulation uptake at the maturity stage;

NRF (%) = Nitrogen accumulation above ground with nitrogen treatment − Nitrogen accumulation above ground with no-nitrogen/Total nitrogen application rate × 100;

NAE = Grain yield with nitrogen treatment − Grain yield with no nitrogen treatment/Total nitrogen application rate.

### Statistical analyses

The experimental data for YY1540 and TYHZ were analyzed statistically using one-way analysis of variance as implemented in SAS 9.1 software (SAS Corp, Cary, NC, USA). Multiple comparisons of the different treatments (three replicates) were analyzed with Duncan’s multiple range test (*p* < 0.05) using SAS 9.1 (SAS Corp.). Graphs were generated using Origin 9.1 (Origin Lab, Northampton, MA, USA).

## Supplementary Information

Supplementary Information

## Data Availability

The data used or analyzed during the current study are available from the corresponding author on reasonable request.

## Abbreviations

TNT: Nitrogen amount transport from stem, sheaths and leaves to panicles
NTE: Apparent nitrogen translocation efficiency of stem, sheaths, and leaves
NCR: Rate of contribution of transferred nitrogen into grains
NMP: Nitrogen dry matter production efficiency
NU_t_E: Nitrogen utilization efficiency
NRF: Apparent nitrogen recovery fraction
NAE: Nitrogen agronomic efficiency
