# Supplementary material for: The nitrogen topdressing mode of indica-japonica and indica hybrid rice are different after side-deep fertilization with machine transplanting
Source: Sci Rep. 2021 Jan 15;11:1494. doi: 10.1038/s41598-021-81295-4 (PMC7810741; doi:10.1038/s41598-021-81295-4)
Supplement: Supplementary file 1 — Supplementary Information [file 41598_2021_81295_MOESM1_ESM.docx]

**The nitrogen topdressing mode of *indica-japonica* and *indica* hybrid rice are different after side*-*deep fertilization with machine transplanting**

Xiaodan Wang, Yaliang Wang*, Yuping Zhang, Jing Xiang, Yikai Zhang, Defeng Zhu, Huizhe Chen*

State Key Laboratory of Rice Biology, China National Rice Research Institute, Hangzhou, Zhejiang 310006, P.R. China

Corresponding authors:

Wang Yaliang, Tel: +86 15356698255, E-mail: [wangyaliang@caas.cn](mailto:wangyaliang@caas.cnm)

Chen Huizhe, Tel: +86 15355460231, E-mail: [chenhuizhe@163.com](mailto:chenhuizhe@163.com)

Table S1. Effect of different nitrogen fertilization mode on rice yield and yield components in 2018

| Cultivar | Treatment | The number of panicles (10^5^ ha^-1^) | The number of spikelet (panicle^-1^) | Seed-setting rate (%) | Grain weight (mg) | Yield (t·ha^-1^) |
| --- | --- | --- | --- | --- | --- | --- |
|  |  |  |  |  |  |  |
| YY1540 | TDP | 15.8±0.1 cd | 350.7±6.6 ab | 80.9±2.3 a | 23.3±0.3 a | 10.5±0.3 bc |
|  | SFT | 15.3±0.2 d | 341.0±32.3 ab | 78.3±4.4 a | 23.1±0.2 abc | 9.5±0.4 d |
|  | B5+T5 | 15.9±0.3 cd | 342.7±9.7 b | 79.7±1.9 a | 22.4±0.1 e | 9.2±0.3 d |
|  | B6+T4 | 16.0±0.6 cd | 331.8±6.5 ab | 81.1±0.9 a | 23.1±0.2 de | 9.7±0.4 cd |
|  | B7+T3 | 16.5±1.0 bc | 327.7±15.4 b | 80.0±1.2 a | 22.8±0.2 cde | 9.9±0.7 cd |
|  | B8+T2 | 15.9±0.1 ab | 322.7±21.7 b | 81.5±0.7 a | 22.8±0.1 cde | 9.5±0.7 d |
|  | B5+P5 | 16.6±0.2 bc | 349.4±10.0 ab | 80.7±1.7 a | 23.2±0.2 ab | 10.9±0.5 ab |
|  | B6+P4 | 17.0±0.4 ab | 346.0±11.5 ab | 81.7±2.6 a | 23.0±0.3 bcd | 11.1±0.4 ab |
|  | B7+P3 | 17.5±0.2 a | 357.5±10.5 a | 80.1±0.3 a | 23.1±0.2 abc | 11.6±0.2 a |
|  | B8+P2 | 11.4±0.3 ab | 347.4±8.5 ab | 81.0±0.8 a | 23.1±0.1 abc | 11.2±0.2 ab |
|  |  |  |  |  |  |  |
| TYHZ | TDP | 19.4±0.3 a | 241.0±10.3 ab | 73.7±1.3 a | 22.9±0.4 ab | 7.9±0.4 ab |
|  | SFT | 19.9±0.4 a | 232.1±4.4 ab | 75.5±1.6 a | 23.4±0.5 a | 8.1±0.2 a |
|  | B5+T5 | 19.8±1.1 a | 231.5±18.6 ab | 73.5±3.3 a | 21.5±0.3 e | 7.2±0.1 ab |
|  | B6+T4 | 19.4±0.7 a | 232.1±12.9 ab | 74.7±2.9 a | 21.6±0.3 e | 7.3±0.6 b |
|  | B7+T3 | 18.8±0.5 a | 239.4±11.8 ab | 74.9±1.4 a | 22.1±0.4 cde | 7.4±0.4 ab |
|  | B8+T2 | 18.9±1.0 a | 233.7±2.2 ab | 75.1±2.0 a | 21.9±0.3 de | 7.3±0.7 b |
|  | B5+P5 | 19.6±0.3 a | 229.5±7.5 b | 74.1±3.0 a | 22.8±0.2 abc | 7.6±0.5 ab |
|  | B6+P4 | 19.0±0.8 a | 237.1±1.3 ab | 75.9±3.7 a | 22.0±0.7 cde | 7.5±0.3 ab |
|  | B7+P3 | 18.6±0.4 a | 250.0±10.8 a | 76.0±1.4 a | 22.4±0.4 bcd | 7.9±0.3 ab |
|  | B8+P2 | 19.4±0.9 a | 226.4±11.8 b | 74.7±1.3 a | 22.1±0.2 cde | 7.2±0.6 b |
|  |  |  |  |  |  |  |
| Cultivar | | 361.16** | 953.31** | 99.76** | 64.63** | 542.88** |
| Treatment | | 1.26 | 2.24** | 0.53 | 10.29** | 6.67** |
| Cultivar×Treatment | | 4.50** | 1.2 | 0.55 | 2.16 | 4.94** |

Total nitrogen rate: 195 kg·ha^-1^. TDP: Traditional nitrogen fertilization with quick-release nitrogen at the 1 days before transplanting (base fertilizer, 40%N), 7 days after transplanting (tillering fertilizer, 30% N) and panicle initiation stage (panicle fertilizer, 30% N); SFT: Control-release nitrogen by single-dose side deep fertilization with machine transplanting (100% N); B5+T5: Controlled-release nitrogen by side deep fertilization machine transplanting (50% N)+ Quick-release nitrogen top dressing at 7days after transplanting (50% N); B6+T4: Controlled-release nitrogen by side deep fertilization machine transplanting (60% N)+ Quick-release nitrogen top dressing at 7days after transplanting (40% N); B7+T3: Controlled-release nitrogen by side deep fertilization machine transplanting (70% N)+ Quick-release nitrogen top dressing at 7days after transplanting (30% N); B8+T2: Controlled-release nitrogen by side deep fertilization machine transplanting (80% N)+ Quick-release nitrogen top dressing at 7days after transplanting (20% N); B5+P5: Controlled-release nitrogen by side-deep fertilization machine transplanting (50% N)+ Quick-release nitrogen top dressing at panicle initiation stage (50% N); B6+P4: Controlled-release nitrogen by side-deep fertilization machine transplanting (60% N)+ Quick-release nitrogen top dressing at panicle initiation stage (40% N); B7+P3: Controlled-release nitrogen by side-deep fertilization machine transplanting (70% N)+ Quick-release nitrogen top dressing at panicle initiation stage (30% N); B8+P2: Controlled-release nitrogen by side-deep fertilization machine transplanting (80% N)+ Quick-release nitrogen top dressing at panicle initiation stage (20% N).
